# The Magnitude and Patterns of Acquired Drug Resistance Mutations and Circulating HIV-1 Subtypes in HIV Patients in Tanzania, a Systematic Review and Meta-Analysis

**DOI:** 10.3390/v17081087

**Published:** 2025-08-06

**Authors:** Shimba Henerico, Christa Kasang, Benson R. Kidenya, Deodatus Sabas, Violet D. Kajogoo, Gert Van Zyl, Wolfgang Preiser, Stephen E. Mshana, Samuel E. Kalluvya

**Affiliations:** 1 Bugando Medical Centre, Mwanza P.O. Box 1370, Tanzania; 2 German Leprosy and Tuberculosis Relief Association (DAHW), Raiffeisenstraße 3, 97080 Würzburg, Germany; 3 Medmissio Würzburg, 97074 Würzburg, Germany; 4 Department of Biochemistry and Molecular Biology, Weill Bugando School of Medicine, Catholic University of Health and Allied Sciences-Bugando, Mwanza P.O. Box 1464, Tanzania; 5 Directorate of Library Services, Muhimbili University of Health and Allied Sciences (MUHAS), Dar es Salaam P.O. Box 65001, Tanzania; 6 Tanzania Diabetes Association (TDA), Dar es Salaam P.O. Box 65201, Tanzania; 7 National Health Laboratory Service (NHLS), Division of Medical Virology, Faculty of Medicine and Health Sciences, Stellenbosch University, Tygerberg, Cape Town 7602, South Africa; 8 Department of Microbiology and Immunology, Weill Bugando School of Medicine, Catholic University of Health and Allied Sciences-Bugando, Mwanza P.O. Box 1464, Tanzania; 9 Department of Internal Medicine, Weill Bugando School of Medicine, Catholic University of Health and Allied Sciences-Bugando, Mwanza P.O. Box 1464, Tanzania

**Keywords:** HIV drug resistance, HIV-1 subtypes, Tanzania, systematic review and meta-analysis

## Abstract

The emergence and spread of HIV drug resistance mutations (DRMs) pose a threat to current and future treatment options. To inform policy, this review aimed to determine the magnitude and patterns of DRMs in patients on ART in Tanzania. A systematic literature search was conducted in MEDLINE through PubMed, Embase, and CINAHL up to December 2024. A total of 9685 HIV patients from 23 eligible studies were analyzed. The prevalence of virological failure in studies that used a threshold of >1000 and >400 copies/mL was 24.83% (95% CI: 17.85–32.53%) and 36.94% (95% CI: 24.79–50.00%), respectively. Major DRMs were observed at 87.61% (95% CI: 76.25–95.91%). A decrease in prevalence was observed in studies conducted from 2019, with a pooled prevalence of 62.15% (95% CI: 31.57–88.33%). The most frequently observed HIV-1 subtypes were subtype C at 36.20% (95% CI: 30.71–41.85%), A1 at 33.13% (95% CI: 28.23–38.20%), and subtype D at 16.00% (95% CI: 11.41–21.12%), while recombinant forms of the virus were observed at 13.29% (95% CI: 9.79–17.17%). The prevalence of DRMs against NRTIs and NNRTIs was significantly high, while that against INSTIs and PIs was low, supporting the continued use of PI- and INSTI-based regimens in Tanzania and the need for continued surveillance of DRMs.

## 1. Introduction

The human immunodeficiency virus (HIV) pandemic remains a significant public health problem, with approximately 39.9 million people reported to be infected globally by the end of 2023 [1]. More than 70% of the global population of people living with HIV (PLHIV) resides in sub-Saharan Africa, with the Eastern and Southern regions being the most heavily affected by the pandemic and accounting for about 52% of the global cases by the end of 2023 [1,2]. The fight against HIV/AIDS continues to be a health priority in many countries, with the use of Antiretroviral (ARV) therapy as a key treatment and prevention strategy. This approach has contributed to reductions in HIV incidence, HIV/AIDS-related morbidity and mortality, and has improved life expectancy in PLHIV [2].

In Tanzania, antiretroviral therapy (ART) was introduced free of charge in 2004 with Stavudine (d4T)-based regimens. However, due to toxicity concerns, d4T was later phased out starting in 2010 and replaced by another thymidine analogue, Zidovudine (AZT), following World Health Organization (WHO) recommendations for public health treatment approach [3]. From 2015, the program transitioned to Tenofovir Disoproxil Fumarate (TDF) as the preferred backbone in both first- and second-line regimens due to its improved safety profile compared with d4T and AZT. [4]. In 2019, the WHO recommended the use of dolutegravir (DTG), a second-generation Integrase Strand Transfer Inhibitor (INSTI), in first- and second-line regimens, in response to increasing levels of transmitted resistance to Non-nucleoside Reverse Transcriptase Inhibitors (NNRTIs) such as Efavirenz (EFV) and Nevirapine (NVP) [5]. A rapid scale-up of ART has been observed in Tanzania from 20% of the about 1.2 million PLHIV in 2010 to approximately 82% of the 1.7 million PLHIV by the end of 2023. Over this period, ARV use has led to notable outcomes, including a 39% reduction in HIV incidence and a 52% reduction in AIDS-related deaths, as reported by UNAIDS (United Republic of Tanzania|UNAIDS). Tanzania is also progressing in achieving the third target of the UNAIDS 95-95-95 goals, whereby 87% of PLHIV know their status, 87% on ART, and 79% of those on ART were virally suppressed by the end of 2023 [1].

Despite these achievements, the emergence and spread of drug-resistant HIV strains threaten to undermine the gains made, as resistances compromise the efficacy of ARV drugs in reducing HIV incidence, HIV-related morbidity, and mortality [6]. Elevated levels of drug resistance mutations (DRMs) have been reported in various populations receiving ART in Tanzania and pose a considerable threat to achieving the desirable treatment outcomes [7,8,9,10]. Understanding national burden and patterns of DRMs is vital for informing policy decisions on current and future therapeutic options, improving practices, and ensuring the proper allocation of the limited resources to devise interventions aimed at effectively curbing the spread of resistant virus strains.

In this review, we aimed to determine the magnitude and patterns of acquired drug resistance mutations, as well as the HIV-1 subtypes circulating in Tanzania.

## 2. Materials and Methods

A systematic literature review was conducted from February to May 2025 following PRISMA standards [11], including original studies that were conducted in Tanzania involving participants receiving ART published in English by the end of December 2024. The systematic review protocol was registered in the International Prospective Register of Systematic Reviews (PROSPERO) on 7 January 2025 under the registration number CRD42025606698.

### 2.1. Search Strategy

**Figure 1:**
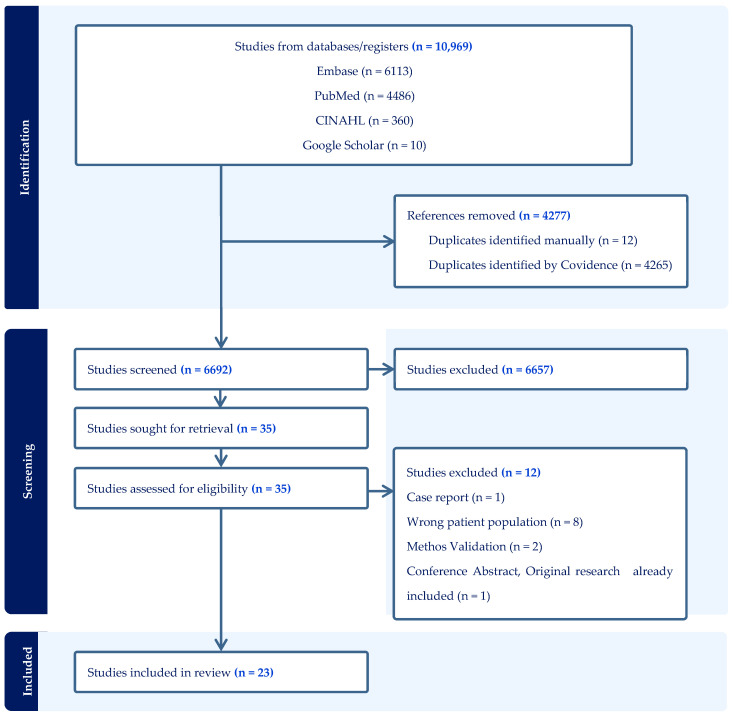
PRISMA flow chart showing the search results and screening process.

The systematic literature search was carried out between February and March 2025 on online databases including MEDLINE through PubMed, Embase, and CINAHL, while Google Scholar was used to identify grey literature and would not be identified by other databases. The concepts “Drug Resistance Mutations”, “Drug Resistance”, “ART resistance”, “HIV-1 Subtypes”, “HIV Subtypes”, “HIV”, and “Tanzania” were used as key concepts alongside related search terms to construct the search strategy. Boolean operators “AND”, “OR”, and “NOT” were used to combine different concepts, similar terms, and exclude studies. Filters and field searches, such as Mesh Terms and truncations, were applied to refine the search. The search strategy was created for PubMed search first, and then it was translated to meet other databases’ requirements. The summary of the search strategies for the databases is provided in Supplementary Materials (Table S1). Each article was reviewed for relevance, and those relevant to our topic were included in this review (Figure 1).

### 2.2. Inclusion and Exclusion Criteria

#### 2.2.1. Inclusion Criteria

Clinical trial, cross-sectional, longitudinal, and case–control studies that were conducted in Tanzania among HIV-infected individuals receiving ART, aiming at reporting the magnitude of drug resistance mutations and/or HIV-1 subtype distributions, were included in this review. Titles and abstracts were screened for relevance, and full texts of potential reports were thereafter reviewed for inclusion.

#### 2.2.2. Exclusion Criteria

Case reports and/or case series and primary studies that did not aim at reporting the magnitude of drug resistance mutations and/or HIV-1 subtype distribution in HIV patients on ART were excluded.

### 2.3. Study Screening

Titles and abstracts of potential studies to be included in this review were independently screened for relevance by S.H. and V.K. Full texts were retrieved and assessed in cases where the information provided in the titles and abstracts was deemed insufficient to make decisions at this stage. A third reviewer (DS) was involved in settling divergencies between the two reviewers.

### 2.4. Data Extraction

A standardized data extraction form created in Microsoft Excel was used to systematically record information from the studies included. The following study characteristics were recorded from each of the included studies: author(s), title of the study, publication year, database used for genotyping, and the year of the study as described by authors (sampling year). Patient characteristics that were recorded were age group (children or adults) as defined in the included studies. Some studies had youth and adolescents as study participants; in this analysis, these groups were all categorized as children. Additional information that was collected was mean or median age and sex.

Outcomes that were recorded were the overall and specific prevalence of drug resistance mutations against Nucleotide/Nucleoside Reverse Transcriptase Inhibitors (NRTIs), Non-Nucleoside Reverse Transcriptase Inhibitors (NNRTIs), Protease Inhibitors (PIs), Integrase Strand-Transfer Inhibitors (INSTIs), and HIV-1 subtypes that were reported. Information from studies that reported stratified data based on different populations was separately extracted based on each stratum’s information. When available, virological failure was recorded and stratified based on the thresholds defined by each study.

### 2.5. Risk of Bias Assessment

The Joanna Briggs Institute (JBI) critical appraisal tools for prevalence studies (https://jbi.global/critical-appraisal-tools, accessed on 2 June 2025) was used to assess the quality of the included studies at an overall JBI score of 50% [12]. This tool was chosen over the ones indicated in our PROSPERO registration since its domains suited the prevalence studies that were included. This process was independently carried out by two reviewers, SH and VDK; conflicts were resolved by discussion involving a third reviewer (DS). The summary for the risk of bias assessment was presented using the Risk-of-Bias VISualization (ROBVIS) tool (https://www.riskofbias.info/welcome/robvis-visualization-tool, accessed on 26 May 2025) [13].

### 2.6. Data Analysis

The main outcomes of this review were the prevalence of acquired drug resistance mutations expressed as a proportion of the total number of mutations to the total number of sequences and HIV-1 subtype distribution expressed in proportion of the total number of a specific subtype to the total number of sequences. Virological failure, when available, was expressed as the total number of individuals with a viral load higher than the set virological failure threshold to the total number of individuals in whom viral load was assessed. The pooled estimates of virological failure, drug resistance mutations, together with their 95% confidence intervals, were determined using Restricted Maximum Likelihood random-effect models. The meta-analysis was performed using Stata 18.0. software (Stata Corp., College Station, TX, USA). We assessed heterogeneity between included studies using the I-squared statistic (I^2^); high heterogeneity was considered where I^2^ > 50% [14]. Subgroup analysis was considered to identify the possible sources of variation whenever high heterogeneity was observed using sampling year, age category, and virological failure thresholds as potential factors. Sensitivity analysis was conducted by systematically omitting one study at a time to assess the robustness of results and explore potential sources of heterogeneity.

Visual inspection of the funnel plot symmetry was used to assess publication bias in the included studies; the Egger’s test was used when publication bias or small study effect was observed by the plot’s asymmetry, and a *p* value < 0.05 indicated significant evidence of publication bias or small study effect [15]. Forest plots were used for visual summarization of the meta-analysis results, illustrating both individual and pooled estimates with their level of accuracy.

## 3. Results

### 3.1. Study Selection

A total of 10,969 articles were obtained from MEDLINE through PubMed, CINAHL, and Embase based on our search strategy. Of these, 6624 articles were excluded at screening, and 12 articles were excluded at the full-text screening stage. Among the 12 studies that were excluded, 1 was a case report, 8 studies were conducted in ART naïve individuals, 2 were method validation studies, and the last one was a conference abstract whose main research article had been included. Twenty-three (23) studies were included in this review; two studies [8,16] reported outcomes separately for adults and children, making 25 studies during the analysis. This information is summarized and illustrated in the PRISMA flow diagram presented in Figure 1.

### 3.2. Risk of Bias in Included Studies

**Figure 2:**
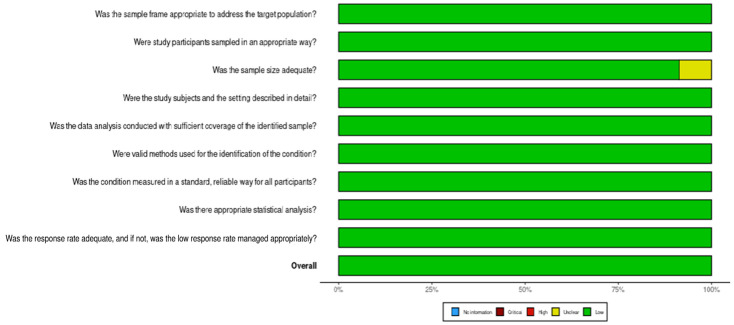
Risk of bias plot.

All the assessment domains of the checklist were sufficiently and appropriately covered by all the included studies. Most of the included studies had a justified sample size (*n* = 21, 91.3%). The summary of the assessed domains is illustrated in Figure 2.

### 3.3. Characteristics of Included Studies

All included studies were observational studies, including a total of 9685 participants on ART, whereby 6677 (68.94%) were women. Eight (34.78%) of included studies involved child participants, whereby a total of 2320 (23.95%) children were enrolled. The characteristics of studies that were included with their respective participants are summarized in Table 1.

### 3.4. Results of the Meta-Analysis

The primary outcome of this study was the magnitude and patterns of drug resistance mutations among PLHIV receiving ART, while the secondary outcome was the HIV-1 subtypes’ distribution in the included studies. Some studies followed participants longitudinally, assessing virological failure as well as DRMs prevalence, and other studies enrolled participants who already had virological failure. We are reporting virological failure in studies in which the outcome was assessed. All the results from our analysis are summarized in Table 2, while individual outcomes are fully described in their respective sub-sections.

### 3.5. Virological Failure

**Figure 3:**
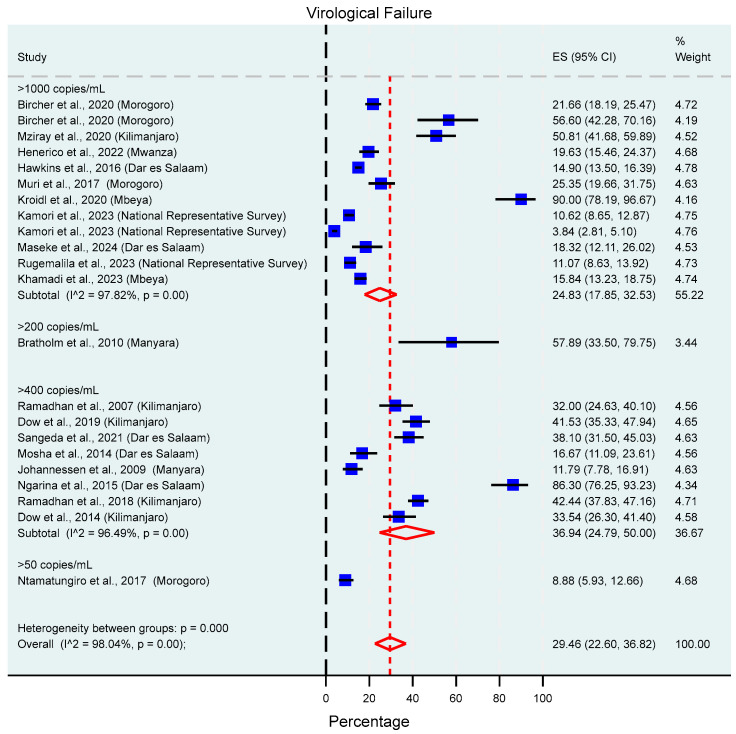
Prevalence of virological failure in HIV patients on ART in Tanzania from 2004 to 2024 based on different virological failure thresholds. The blue squares present individual study effect size; the size of the square shows the weight of the study while the whiskers show the confidence intervals. The pooled effect estimate is presented by the red diamond.

Virological failure was reported in 22 of the included studies, and different virological failure thresholds were used in different studies. The pooled prevalence of virological failure was 29.46% (95% CI: 22.60–36.82%, I^2^ = 98.04%), and subgroup analysis using the different virological failure thresholds applied in the included studies yielded a prevalence of 24.83% (95% CI: 17.85–32.53%, I^2^ = 97.82%) and 36.94% (95% CI: 24.79–50.00%, I^2^ = 96.49%) in studies that used thresholds of >1000 copies/mL and >400 copies/mL, respectively (Figure 3). Meanwhile, two individual studies reported a prevalence of 57.89% (95% CI: 33.50–79.75%) and 8.88% (95% CI: 5.93–12.66%), when thresholds of >200 copies/mL and >50 copies/mL were, respectively, applied. This information is summarized in Figure 3. Further subgroup analyses showed that virological failure thresholds, the region in which the study was conducted, and sampling year contributed to the observed high heterogeneity between studies (all *p*-values < 0.01), and no significant difference was observed between age categories. This information is available in the Supplementary Materials (Figures S1–S3). Sensitivity analysis conducted by omitting one study at a time showed that the results are robust with a summary estimate of 29.7% (20.7–38.7%, *p* < 0.01). The sensitivity analysis results for virological failure are summarized in Supplementary Materials Table S2.

### 3.6. Acquired Drug Resistance Mutations

**Figure 4:**
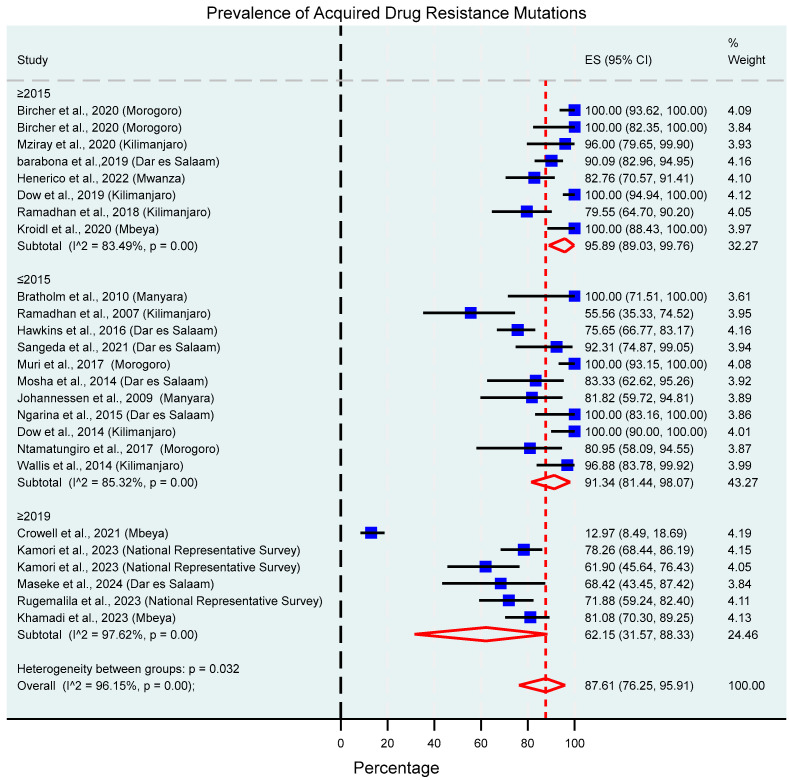
Prevalence of acquired drug resistance mutations in viremic HIV patients whose samples were successfully sequenced in Tanzania between 2004 and 2024, based on the study’s sampling year. The blue squares present individual study effect size; the size of the square shows the weight of the study while the whiskers show the confidence intervals. The pooled effect estimate is presented by the red diamond.

**Figure 5:**
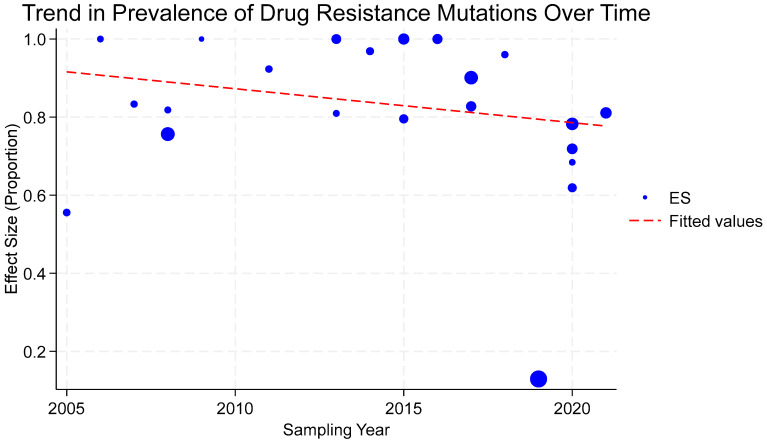
Prevalence of acquired drug resistance mutations in viremic HIV patients whose samples were successfully sequenced in Tanzania between 2004 and 2024, by the year in which the study was conducted (sampling year). Studies are represented in bubbles; the size of the bubbles is proportional to the study size, and the trend line is predicted prevalence.

The overall prevalence of acquired drug resistance mutations in the participants with virological failure whose samples were successfully genotyped was 87.61% (95% CI: 76.25–95.91%, I^2^ = 96.15%), and subgroup analysis showed a significant decline over time in the prevalence of DRMs, especially in studies that were conducted from beyond 2019, with a prevalence of 62.15% (95% CI: 31.57–88.33%, I^2^ = 97.62%), as shown in Figure 4 and Figure 5. Additional subgroup analyses revealed significantly different proportions across different viral load thresholds (*p* = 0.0317), regions (*p* = 0.03), and age categories (*p* = 0.012). This information is illustrated in Supplementary Materials Figures S4–S6.

#### 3.6.1. Publication Bias Assessment for Drug Resistance Mutations

**Figure 6:**
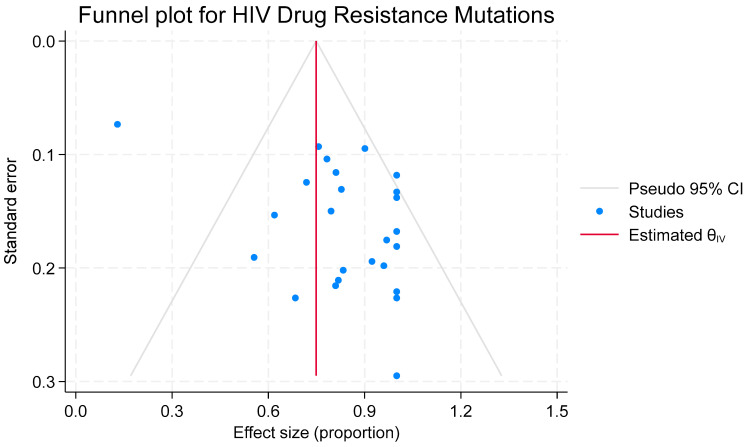
Bias assessment plot for studies reporting the prevalence of drug resistance mutations.

The funnel plot symmetry indicated signs of potential risk of publication bias or small study effect (Figure 6). The Egger’s test showed statistical evidence that small-study or selective reporting had no effect on the DRMs estimate (β_1_ = 1.77, SE = 0.946, z = 1.87, *p* = 0.0612). Additionally, results of a leave-one-out sensitivity analysis show a robust pooled prevalence with a summary estimate of 81.7% (72.0–91.4%, *p* < 0.01. The results summary for DRM sensitivity analysis is in Supplementary Materials Table S3.

#### 3.6.2. Drug Resistance Mutations Against Reverse Transcriptase Inhibitors

##### NRTIs

**Figure 7:**
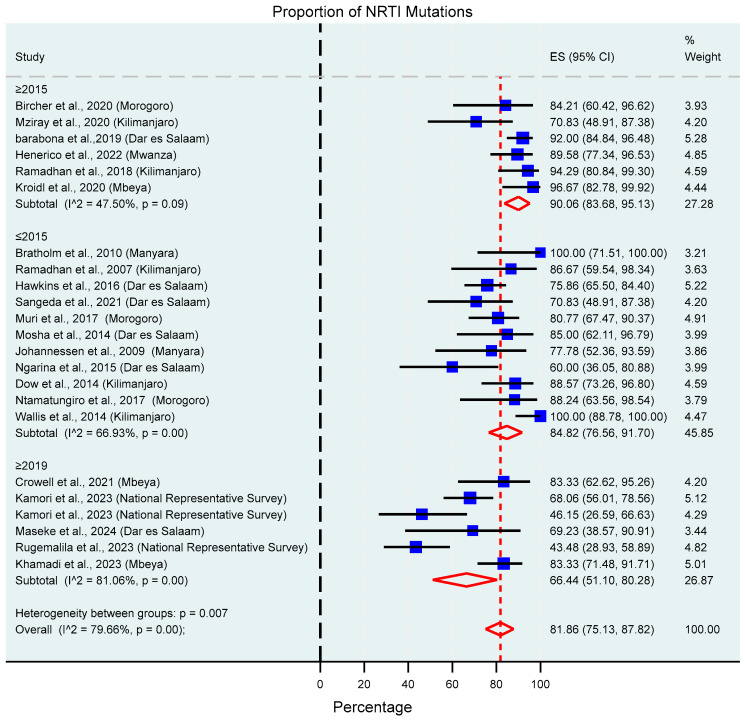
Proportion of NRTI mutations based on the study’s sampling year. The blue squares present individual study effect size; the size of the square shows the weight of the study while the whiskers show the confidence intervals. The pooled effect estimate is presented by the red diamond.

The proportion of drug NRTI mutations was 81.86% (95% CI: 75.13–87.82%, I^2^ = 79.66%), as shown in Figure 7. The prevalent NRTI mutations were M184V/I 92.56% (95% CI: 84.38–98.23%, I^2^ = 86.42%), T215Y/F/S/V 40.69% (95% CI: 3.71–48.90%, I^2^ = 47.46%), D67G/N 36.75% (95% CI: 29.24–44.57%, I^2^ = 41.37%), K70R/E/S 32.8% (95% CI: 24.9–41.1%, I^2^ = 52.6%), K219Q/R/E/N 27.50% (95% CI: 17.52–38.62%, I^2^ = 67.44%), M41L 24.97% (95% CI: 16.39–34.54%, I^2^ = 66.97%), and L210W 14.34% (95% CI: 9.58–19.75, I^2^ = 0.00%). In studies where TAMs were grouped, a prevalence of 42.21% (95% CI: 22.01–63.73%, I^2^ = 87.61%) was observed. This information is summarized in Table 2.

##### NNRTIs

**Figure 8:**
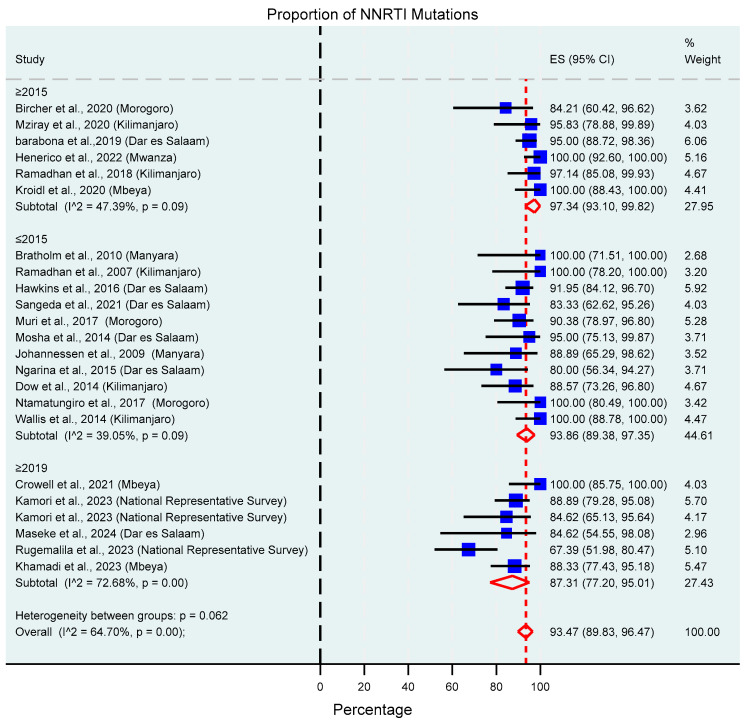
Proportion of NNRTI mutations based on the study’s sampling year. The blue squares present individual study effect size; the size of the square shows the weight of the study while the whiskers show the confidence intervals. The pooled effect estimate is presented by the red diamond.

The prevalence of NNRTI mutations was 93.47% (95% CI: 9.83–96.47%, I^2^ = 64.70%) as shown in Figure 8. The most prevalent NNRTI mutations were K103N/S 48.98% (95% CI: 44.04–53.94%, I^2^ = 21.27%), Y181C/I/V 30.27% (95% CI: 24.47–36.37%, I^2^ = 46.57%), G190A/S/E/Q 26.65% (95% CI: 21.70–31.88%, I^2^ = 26.18%), and H221Y 20.34% (95% CI: 11.39–30.82%, I^2^ = 20.13%).

#### 3.6.3. Other Mutations

PI mutations were reported in six studies and were observed at 7.33% (95% CI: 3.41–12.37%, I^2^ = 50.88%), while mutations against INSTI reported in four studies were observed at 6.61% (95% CI: 3.37–10.68%, I^2^ = 0.0%). This information is summarized in Supplementary Materials Figures S7 and S8.

### 3.7. HIV-1 Subtypes

**Figure 9:**
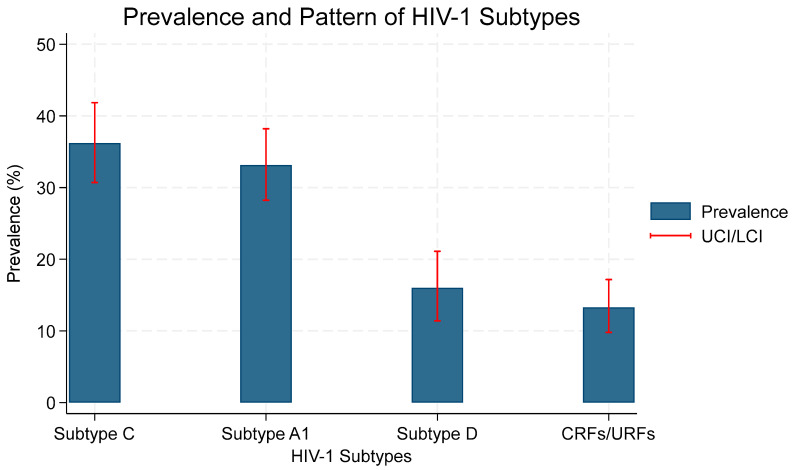
Pooled prevalence of HIV-1 subtypes, with their lower and upper confidence intervals (LCI and UPCI, respectively).

The most prevalent HIV-1 subtype was subtype C 36.20% (95% CI: 30.71–41.85%, I^2^ = 71.62%), A1 33.13% (95% CI: 28.23–38.20%, I^2^ = 65.69%), followed by subtype D 16.00% (95% CI: 11.4–21.1%, I^2^ = 75.96%), while recombinant forms of the virus were observed at 13.29% (95% CI: 9.77–17.17%, I^2^ = 64.98%). This information is illustrated in Figure 9.

## 4. Discussion

Virological failure was assessed and reported in 22 of the included studies, and the pooled prevalence was 29.46%. A slight increase in the proportion was observed as lower virological failure thresholds were used with 24.83% and 36.94% in studies that used a threshold of >1000 copies/mL and >400 copies/mL, respectively. Subgroup analyses highlighted a peak between 2015–2019 before declining afterwards, indicating the advantages of the pragmatic transition to Dolutegravir-based regimens in the country, whereby virological suppression was achieved in about 89% of PLHIV on ART. The observed prevalence for studies using the >1000 copies/mL threshold is consistent with Tanzania’s progress report towards the third target on the 95%, 95%, 95% goal where about 79% of PLHIV were virally suppressed by the end of 2023 [1] and higher than that reported in a review that was assessing virological failure in East Africa between 2016–2023 which reported a prevalence of 19.4% [35]. Our findings are also higher than those reported in the 2021 Tanzania national representative survey, with a virological failure rate reported at 3.9% and 10.9% in adults and children, respectively [36]. The funnel plot and Egger’s test suggest the presence of publication or small study effects, which might have inflated our pooled effect size.

The country may consider reviewing and lowering the virological failure threshold from the >1000 copies/mL, since the established infrastructures support routine viral load monitoring, detecting as low as 20 copies/mL. Additionally, this is supported by the fact that drug resistance mutations have been detected in patients with fewer than 1000 copies/mL. Hence, early treatment failure detection helps minimize the risks associated with treatment failure, including an increase in viral load, further accumulation of drug resistance mutations, which complicate future treatment options, transmission of resistance strains of the virus, and other adverse outcomes such as co-morbidities and mortality.

This review reports a high pooled prevalence of acquired DRMs (87.61%) among individuals with virological failure on ART whose samples were successfully genotyped. The high heterogeneity between studies is explained by the differences in the sampling year, different virological failure thresholds, regions in which the studies were conducted and age category difference whereby children had the highest prevalence.

High prevalence was also observed against the commonly used classes of ARV drugs, NRTIs (81.86%), as well as NNRTIs (93.47%). Resistance to PIs and INSTIs was low, although slightly higher than compared with the national representative survey that was conducted in 2021 [36]. A declining trend in drug resistance mutation prevalence was observed from 2019 onwards and is strongly linked with the introduction of Dolutegravir as an anchor drug in first- and second-line regimens. The transition has led to improved virological suppression rates, for instance, those observed in the national representative surveillance; 96.1% and 89.1% in adults and children, respectively [36]. Therefore, the high genetic barrier to resistance possessed by DTG and improved virological suppression altogether reduce the emergence of drug resistance mutations. This can explain why the prevalence from the national representative survey is slightly lower as well.

High drug resistance mutation proportions were observed in participants where lower levels of the virological failure thresholds were used (90.8%, 95% CI: 80.6–97.8%), with a threshold of as low as 400 cp/mL, as well as two individual studies that used thresholds of as low as 200 cp/mL [18] and 50 cp/mL [30], respectively (Supplementary Figure S1). This finding highlights the need to start closely monitoring low-level viremia, as it may indicate adherence challenges, suboptimal treatment response, or emerging drug resistance. The surveillance of low-level viremia will allow timely interventions to attain sustained long-term treatment outcomes.

The most frequently observed NRTI mutation was M184V/I, which confers high-level resistance to 3TC and FTC. The high frequency of this mutation is explained by the extensive use of 3TC- and FTC in both first- and second-line ART regimens in the country to date. Despite their resistance profiles, these ARV drugs are still being used because of the replication disadvantage and increased susceptibility to other NRTIs such as AZT and TDF brought about by the M184V mutation [37,38]. Thymidine Analogue Mutations (TAMs), which reduce in vitro susceptibility to AZT and d4T, were also prevalently observed. This can be attributed to Tanzania’s initial ART regimens being d4T-based, followed by AZT, both of which are thymidine analogues. This is also evidenced by the observed increasing trend in the proportions of TAMs through these transitions in ART regimens (Table 2) [38]. Thymidine analogues have been the mainstay of our national ART program since it started in 2004 with Stavudine (d4T), and was then replaced with AZT, which is still being used to date [38]. The use of both d4T and AZT is associated with the emergence of TAMs [39,40]. The observed low prevalence of K65R despite the vast use of TDF in our ART program since 2015 is attributed to the existence of bidirectional phenotypic antagonism between K65R and TAMs [41], brought about by the existence of multiple TAMs due to extensive use of thymidine analogues in the country.

It was observed that more than 90% of all successfully genotyped samples had at least one NNRTI mutation with K103N/S Y181C/I/V, G190A/S/E/Q, and H221Y being the prevalent mutations, which confer reductions in EFV and NVP susceptibility. The presence of Y181C in abundance poses a threat to the future therapeutic options because it confers reduced susceptibility to second-generation NNRTIs such as Etravirine (ETR) and Rilpivirine (RPV), provided that cross-resistance between first- and second-generation NNRTIs has been observed in our settings [10].

A low proportion of PI mutations was observed, and this is in line with the fact that only 3% of PLHIV on ART are on second-line regimens [42]. This finding supports the wide use of PIs in second-line regimens, as recommended by the national guidelines [38]. The proportion of INSTIs mutations observed, though low, poses a concern given the fact that INSTIs, in particular Dolutegravir (DTG), were introduced in Tanzania in 2019 and underscores the need for continued surveillance of INSTI mutations to warrant achieving treatment goals. The observed findings are consistent with other studies that have been conducted in neighboring countries such as Rwanda, Mozambique, and Zambia, where high Reverse transcriptase (RT) mutation rates and low PI and INSTI mutation rates have been reported [43,44,45]. The findings support the use of TDF/TAF and DTG-based first-line regimens and PI whenever treatment failure is experienced in Tanzania, as recommended by the National Integrated Guideline for management of HIV, STI, and Viral Hepatitis [38].

Among studies that reported HIV-1 subtypes, subtype C was the most prevalent, followed by subtypes A1 and D. Circulating recombinant forms (CRFs) and unique recombinant forms (URFs) were also observed, accounting for 13.29% of the reported sequences, with higher proportions noted in more recent years [7,16,26,27,33,34].

## 5. Conclusions

This pooled analysis of studies conducted in Tanzania reports a high prevalence (87.61%) of drug resistance mutations in PLHIV on ART, particularly against NRTIs and NNRTIs. The decline in DRM prevalence following the introduction of INSTIs-based regimes since 2019 is promising and warrants continued monitoring. Low prevalences of drug resistance mutations against PIs and INSTIs were observed, while HIV-1 subtypes C, A1, D, and circulating as well as unique recombinant forms have consistently been reported. These findings support the use of PI- and INSTI-based regimens in Tanzania. Additionally, the findings solidify the use of TDF/TAF-based first-line regimens, AZT-based regimens whenever treatment failure is experienced, and warrant the need for continued surveillance of drug resistance mutations in PLHIV on ART.

## 6. Limitations

This review provides fundamental contextual benchmarks on the magnitude and patterns of HIV drug resistance mutations in Tanzania for over a period of 20 years. However, it has some limitations that we ought to acknowledge. The generalizability of INSTI and PI resistance results is limited by the small sample size in the included studies.

## Figures and Tables

**Table 1:** Characteristics of included studies.

	Author	Sampling Year	Region	Participants (n)	Age Category	Mean/Median Age	Female (%)	VF Threshold (Copies/mL)	VF (%)	Genotyped	Successful Genotyping (%)	Genotyping Database	Prevalence of DRMs (%)					HIV-1 Subtypes (%)			
													Overall	NRTI	NNRTI	PI	INSTI	Subtype A1	Subtype C	Subtype D	CRFs/URFs
1.	Bircher et al. [8]	2005–2016	Morogoro	517	Adults	41 (33–49)	60.8	1000	22	56	-	HIV dB	50	82		1	-	31	47	12	11
				53	Children	8 (5–10)	37.7	1000	57	19	70	HIV dB	63	84	84	-	-	-	-	-	-
2.	Mziray et al. [17]	2017–2018	Kilimanjaro	124	Adults	45 (35–52)	66.1	1000	51	25	96	HIV dB	96	68	92	8	-	36	48	8	8
3.	Bratholm et al. [18]	2009	Manyara	19	Children	5 (3–8)	42.1	200	58	11	100	HIV dB	58	58	58	-	-	26	16	11	5
4.	barabona et al. [7]	2017	Dar es Salaam	166	Adults	-	-	400	100	111	70	HIV dB	90	92	95	13	-	45	31	8	16
5.	Henerico et al. [10]	2013–2017	Mwanza	326	Adults	43.5	66	1000	20	58	91	HIV dB	86	77	86	-	-	36	34	27	7
6.	Ramadhan et al. [19]	2005	Kilimanjaro	150	Adults	41 (19–69)	63	400	32	27	77	-	56	87	100	-	-	37	26	37	-
7.	Dow et al. [20]	2013–2015	Kilimanjaro	280	Children	16.7 (2.8)	54.4	400	42	71	100	HIV dB	100	-	-	-	-	54	21	20	6
8.	Hawkins et al. [9]	2006–2008	Dar es Salaam	2403	Adults	37 (32–43)	70	1000	15	115	71	HIV dB	76	57	70	-	-	27	38	28	6
9.	Sangeda et al. [21]	2010–2011	Dar es Salaam	210	Adults	34.5 (32–42)	55.6	400	23	26	54	HIV dB	92	70	83	-	-	65	35	27	31
10.	Muri et al. [22]	2013	Morogoro	213	Children	11 (7.5–14.4)	43	1000	25	52	92	-	90	81	90	-	-	33	44	21	2
11.	Mosha et al. [23]	2007	Dar es Salaam	150	Adults	-	-	400	17	24	96	HIV dB	83	71	79	-	-	38	29	17	17
12.	Johannessen et al. [24]	2018	Manyara	218	Adults	35 (29–43)	74.5	400	12	22	96	HIV dB	82	78	89	-	-	14	32	36	9
13.	Ngarina et al. [25]	2004–2006	Dar es Salaam	73	Adults	29 (25–32)	100	400	86	20	56	-	34	60	90	-	-	40	20	5	10
14.	Crowell et al. [26]	2013–2019	Mbeya	31	Adults	-	-	1000	-	185	-	HIV dB	77	65	77	3	-	23	54	6	15
15.	Ramadhan et al. [27]	2004–2015	Kilimanjaro	450	Adults	-	67.1	400	42	44	92	HIV dB	80	94	97	-	-	25	18	18	39
16.	Kroidl et al. [28]	2013–2015	Mbeya	356	Adults	-	41.3	1000	90	30	67	HIV dB	100	97	100	-	-	30	63		7
17.	Dow et al. [29]	2012–2013	Kilimanjaro	54	children	12 (9.3–15.3)	52	400	27	35	65	HIV dB	65	89	89	-	-	35	14	31	17
18.	Ntamatungiro et al. [30]	2013	Morogoro	304	Adults	41 (35–48)	70.4	50	9	21	78	-	81	88	100	-	-	33	29	33	5
19.	Kamori et al. [16]	2020	A national representative survey	866	Children	10 (3.6)	58.7	1000	67	92	100	HIV dB	78	53	70	8	5	30	41	7	22
				1173	Adults	38 (13.8)	37.8	1000	33	42	96	HIV dB	58	27	49	9	7	36	45	0	19
20.	Maseke et al. [31]	2020	Dar es Salaam	131	Children	15 (13–18)	55.7	1000	18	19	79	HIV dB	68	69	85	-	-	-	-	-	-
21.	Wallis et al. [32]		Kilimanjaro	32	Adults	39 (22–60)	59	1000	-	32	74	HIV dB	97	97	97	-	-	-	-	-	-
22.	Rugemalila et al. [33]		Dar es Salaam	578	Children	13 (11–14)	55.1	1000	11	64	100	HIV dB	72	44	68	3	6	30	44	4	25
23.	Khamadi et al. [34]	2019–2021	Mbeya	707	Children	12 (9–16)	54	1000	16	74	63	HIV dB	81	68	72	1	4	23	49	7	17

**DRMs**: Drug Resistance Mutations, **VF**: Virological Failure, **NRTIs**: Nucleos(t)ide Reverse Transcriptase Inhibitors, **NNRTIs**: Non-Nucleoside Reverse Transcriptase Inhibitors, **PIs**: Protease Inhibitors, **INSTIs**: Integrase Strand Transfer Inhibitors, **CRFs**: Circulating Recombinant Forms, **URFs**: Unique Recombinant Forms, **HIV dB**: Stanford University HIV Drug Resistance Database, **-**: Not Reported.

**Table 2:** Results Summary.

Variable		Number of Studies	Prevalence% (95% CI)	Heterogeneity (%)	*p*-Value
**Virological Failure**					
	**Overall Prevalence**	22	29.46 (22.60–36.82)	98.04	<0.01
	**Virological Failure Thresholds**				
	>1000 copies/mL	12	24.83 (17.85–32.53)	97.82	<0.01
	>400 copies/mL	8	36.94 (24.79–50.00)	97.8	<0.01
	>200 copies/mL	1	57.89 (33.50–79.75)	NA	NA
	>50 copies/mL	1	8.88 (5.93–12.66)	NA	NA
	**Region**				
	Morogoro	3	25.45 (13.39–39.78)	95.54	<0.01
	Kilimanjaro	5	39.99 (34.44–45.68)	71.47	<0.01
	Manyara	2	13.90 (9.51–18.88)	NA	NA
	Dar es salaam	5	33.54 (15.62–54.30)	98.8	<0.01
	Mwanza	1	19.63 (15.46–24.37)	NA	NA
	Mbeya	2	19.80 (16.99–22.75)	NA	NA
	Multi-regional	3	8.11 (3.62–14.17)	NA	NA
**Drug Resistance Mutations**					
	Overall Prevalence	25	87.61 (76.25–95.91)	96.15	<0.01
	**Sampling Period**				
	≤2015 (Stavudine era)	11	91.34 (81.44–98.07)	85.32	<0.01
	≥2015 (Tenofovir era)	7	95.89 (89.03–99.76)	83.49	<0.01
	≥2019 (Tenofovir + Dolutegravir era)	5	62.15 (31.57–88.33)	97.62	<0.01
	**Virological Failure Thresholds**				
	>1000 copies/mL	14	84.80% (66.57–96.98)	97.34	<0.01
	>400 copies/mL	9	90.78 (80.58–97.75)	85.89	<0.01
	>200 copies/mL	1	100.00 (71.51–100.00)	NA	NA
	>50 copies/mL	1	80.95 (58.09–94.55)	NA	NA
	**ARV Classes**				
	NRTIs	23	81.86 (75.13– 87.82)	79.66	<0.01
	NNRTIs	23	93.47 (89.83–96.47)	64.70	<0.01
	PIs	6	7.33 (3.41–12.37)	50.88	0.07
	INSTIs	4	6.61 (3.37–10.68)	0.00	0.78
	**NRTI DRMs**				
	M184V/I	20	92.6 (84.4–98.2)	86.42	<0.01
	≤2015	10	92.92 (77.43–100.00)	90.33	<0.01
	≥2015	6	94.07 (84.26–99.68)	75.36	<0.01
	≥2019	4	88.85 (62.61–100.00)	87.16	<0.01
	T215Y/F/S/V	13	40.69 (32.71–48.90)	47.46	0.03
	≤2015	6	30.30 (21.77–39.50)	14.29	.032
	≥2015	4	48.28 (37.66–58.98)	26.01	0.26
	≥2019	3	53.21 (32.71–48.90)	0.00	NA
	D67GN	12	36.75 (29.24–44.57)	41.37	0.07
	≤2015	5	30.73 (15.51–48.21)	71.99	0.01
	≥2015	4	42.58 (33.72–51.67)	0.00	0.89
	≥2019	3	38.63 (24.86–53.27)	NA	NA
	K70R/E/S	13	32.80 (24.92–41.14)	52.59	0.01
	≤2015	5	33.18 (19.27–48.61)	63.50	0.03
	≥2015	5	33.36 (19.83–48.33)	66.50	0.02
	≥2019	3	31.54 (17.40–47.41)	NA	NA
	K219Q/R/E/N	10	27.50 (17.52–38.62)	67.44	<0.01
	≤2015	5	18.88 (6.27–35.47)	73.97	<0.01
	≥2015	3	38.57 (28.55–49.07)	NA	NA
	≥2019	2	30.80 (14.46–49.64)	NA	NA
	M41L	13	24.97 (16.39–34.54)	66.97	<0.01
	≤2015	6	13.34 (7.66–20.08)	0.00	0.59
	≥2015	4	30.75 (19.46–43.24)	48.39	0.12
	≥2019	3	44.52 (19.49–70.96	NA	NA
	L210W	10	14.34 (9.58–19.75)	0.00	0.60
	≤2015	5	9.93 (4.03–17.53)	0.00	0.52
	≥2015	3	16.33 (8.58–25.71)	NA	NA
	≥2019	2	22.09 (10.06–36.75)	NA	NA
	T69D	5	12.94 (6.43–20.89)	0.00	0.52
	≤2015	3	11.43 (4.26–20.86)	NA	NA
	≥2015	1	17.65 (3.80–43.43)	NA	NA
	≥2019	1	22.22 2.81–60.01)	NA	NA
	L74V/I/E	6	9.91 (5.10–15.81)	0.00	0.86
	≤2015	2	9.25 (0.84–22.84)	NA	NA
	≥2015	3	11.54 (5.38–19.31)	NA	NA
	≥2019	1	5.00 (0.13–24.84)	NA	NA
	K65R	8	8.15 (4.20–13.04)	29.33	0.19
	≤2015	3	4.15 (0.86–9.05)	NA	NA
	≥2015	4	13.01 (7.32–19.83)	0.38	0.39
	≥2019	1	5.00 (0.86–24.87)	NA	NA
	V75M	4	8.15 (1.49–18.30)	62.57	0.05
	≤2015	2	2.87 (0.00–9.88)	NA	NA
	≥2015	2	13.66 (6.56–22.58)	NA	NA
	TAMs	7	42.21 (22.01–63.73)	87.61	<0.01
	≤2015	5	38.00 (12.67–67.04)	89.55	<0.01
	≥2015	2	46.69 (34.0–59.49)	NA	NA
	**NNRTI DRMs**				
	K103N/S	18	49.98 (44.04–53.94)	21.27	0.20
	≤2015	9	50.04 (43.41–56.67)	0.00	0.52
	≥2015	6	48.82 (41.96–55.70)	0.00	0.76
	≥2019	4	44.83 (25.39–65.06)	76.68	<0.01
	Y181C/I/V	18	30.27 (24.47–36.37)	46.57	0.02
	≤2015	9	35.10 (26.94–43.67)	33.46	0.15
	≥2015	6	22.74 (17.15–28.82)	0.00	0.90
	≥2019	3	40.04 (14.49–68.56)	NA	NA
	G190A/S/E/Q	17	26.65 (21.70–31.88)	26.18	0.15
	≤2015	9	28.20 (19.89–37.23)	40.04	0.10
	≥2015	6	27.77 (21.75–34.18)	0.40	0.41
	≥2019	2	13.85 (3.51–28.23)	NA	NA
	H221Y	5	20.34 (11.39–30.82)	20.13	0.29
	≤2015	2	24.08 (9.47–42.07)	NA	NA
	≥2015	2	13.99 (5.76–24.62)	NA	NA
	≥2019	1	45.45 (16.75–76.62)	NA	NA
	E138K/A/Q/G	11	18.65 (13.41–24.45)	27.65	0.18
	≤2015	4	19.24 (11.30–28.47)	0.00	0.66
	≥2015	5	15.87 (10.68–21.79)	0.00	0.54
	≥2019	2	32.12 (18.25–47.59)	NA	NA
	A98G	6	15.58 (7.92–24.92)	51.58	0.07
	≤2015	2	12.99 (5.10–23.30)	NA	NA
	≥2015	3	15.84 (3.88–32.93)	NA	NA
	≥2019	1	27.27 (6.02–60.97)	NA	NA
	K101E/P	10	15.04 (10.67–19.93)	14.65	0.31
	≤2015	4	15.26 (9.19–22.35)	8.37	0.35
	≥2015	4	16.99 (10.05–25.16)	22.74	0.27
	≥2019	2	7.28 (0.27–19.53)	NA	NA
	V106A/M	6	14.31 (8.58–21.02)	0.00	0.56
	≤2015	2	12.50 (2.00–28.12)	NA	NA
	≥2015	3	14.52 (7.53–23.06)	NA	NA
	≥2019	1	16.67 (4.74–37.38)	NA	NA
	V179L/D/E	5	14.77 (2.87–32.14)	77.45	<0.01
	≤2015	1	11.76 (1.45–36.44)	NA	NA
	≥2015	2	4.88 (0.32–12.74)	NA	NA
	≥2019	2	35.23 (20.92–50.88)	NA	NA
	V108I	7	11.48 (6.51–17.42)	24.06	0.25
	≤2015	5	12.14 (4.69–21.82)	46.32	0.11
	≥2015	2	12.45 (5.40–21.52)	NA	NA
	Y188L/C/H/F	6	10.53 (4.48–18.32)	48.03	0.09
	≤2015	3	16.29 (1.55–39.49)	NA	NA
	≥2015	3	7.92 (2.71–15.01)	NA	NA
	≥2019				
	K238T/N	4	9.36 (4.22–15.92)	0.00	0.64
	≤2015	2	6.49 (1.03–14.93)	NA	NA
	≥2015	2	12.98 (4.79–23.84)	NA	NA
	≥2019				
	L100I	4	5.62 (1.39–11.70)	0.00	0.62
	≤2015	1	11.76 (1.46–36.44)	NA	NA
	≥2015	2	4.88 (0.32–12.74)	NA	NA
	≥2019	1	4.17 (0.11–21.12)	NA	NA
	M230L	4	5.00 (0.59–11.98)	0.00	0.94
	≤2015	2	6.76 (0.00–20.26)	NA	NA
	≥2015	1	4.35 (0.11–21.95)	NA	NA
	≥2019	1	4.17 (0.11–21.12)	NA	NA
	P225H	5	4.22 (1.39–8.09)	0.00	0.46
	≤2015	2	2.43 (0.03–7.03)	NA	NA
	≥2015	2	8.43 (2.10–17.62)	NA	NA
	≥2019	1	4.17 (0.11–21.12)	NA	NA
**HIV-1 Subtypes**					
	Subtype C	22	36.20 (30.71–41.85)	71.62	<0.01
	Subtype A1	22	33.13 (28.23–38.20)	65.69	<0.01
	Subtype D	20	16.00 (11.41–21.12)	75.96	<0.01
	CRFs/URFs	21	13.29 (9.77–17.17)	64.98	<0.01

**DRMs**: Drug Resistance Mutations, **NRTIs**: Nucleos(t)ide Reverse Transcriptase Inhibitors, **NNRTIs**: Non-Nucleoside Reverse Transcriptase Inhibitors, **PIs**: Protease Inhibitors, **INSTIs**: Integrase Strand Transfer Inhibitors, **CRFs**: Circulating Recombinant Forms, **URFs**: Unique Recombinant Forms.

## Data Availability

Data supporting the findings of this study are available from the corresponding author upon request.

## Supplementary Materials

The following supporting information can be downloaded at https://www.mdpi.com/article/10.3390/v17081087/s1, Figure S1: Prevalence of virological failure based on age category, Figure S2: Prevalence of virological failure based on regions, Figure S3: Prevalence of virological failure based on sampling years, Figure S4: Prevalence of drug resistance mutations based on different virological failure thresholds as defined by the included studies, Figure S5: Prevalence of drug resistance mutations based on age categories, Figure S6: Prevalence of drug resistance mutations based on the region where the study was conducted, Figure S7. Proportion of PI mutations, Figure S8. Prevalence of INSTI mutations. Table S1: Literature search strategies for the different databases, Table S2: Sensitivity analysis results for virological failure. Table S3: Sensitivity analysis results for drug resistance mutations.

## Abbreviations

The following abbreviations are used in this manuscript:

ART	Antiretroviral Therapy
ARV	Antiretroviral
DRMs	Drug Resistance Mutations
INSTIs	Integrase Strand Transfer Inhibitors
NNRTIs	Non-Nucleoside Reverse Transcriptase Inhibitors
NRTIs	Nucleotide/Nucleoside Reverse Transcriptase Inhibitors
PLHIV	People Living with HIV
