# Supplementary material for: The Magnitude and Patterns of Acquired Drug Resistance Mutations and Circulating HIV-1 Subtypes in HIV Patients in Tanzania, a Systematic Review and Meta-Analysis
Source: Viruses. 2025 Aug 6;17(8):1087. doi: 10.3390/v17081087 (PMC12390731; doi:10.3390/v17081087)
Supplement: Supplementary file 1 [file viruses-17-01087-s001.zip › viruses-3765654-supplementary.pdf]

Figures

Subgroup analyses for Virological Failure

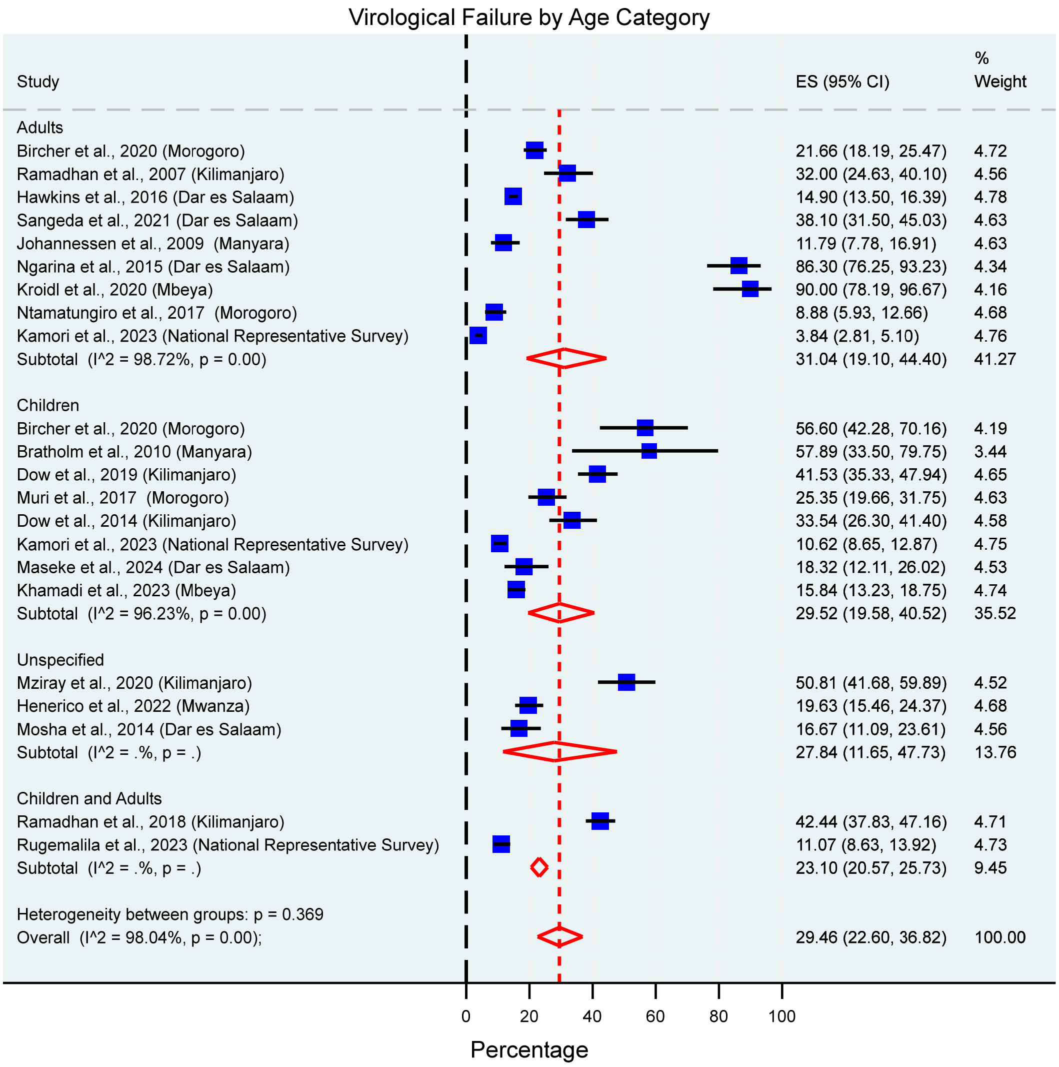

Figure S1. Prevalence of virological failure based on age category

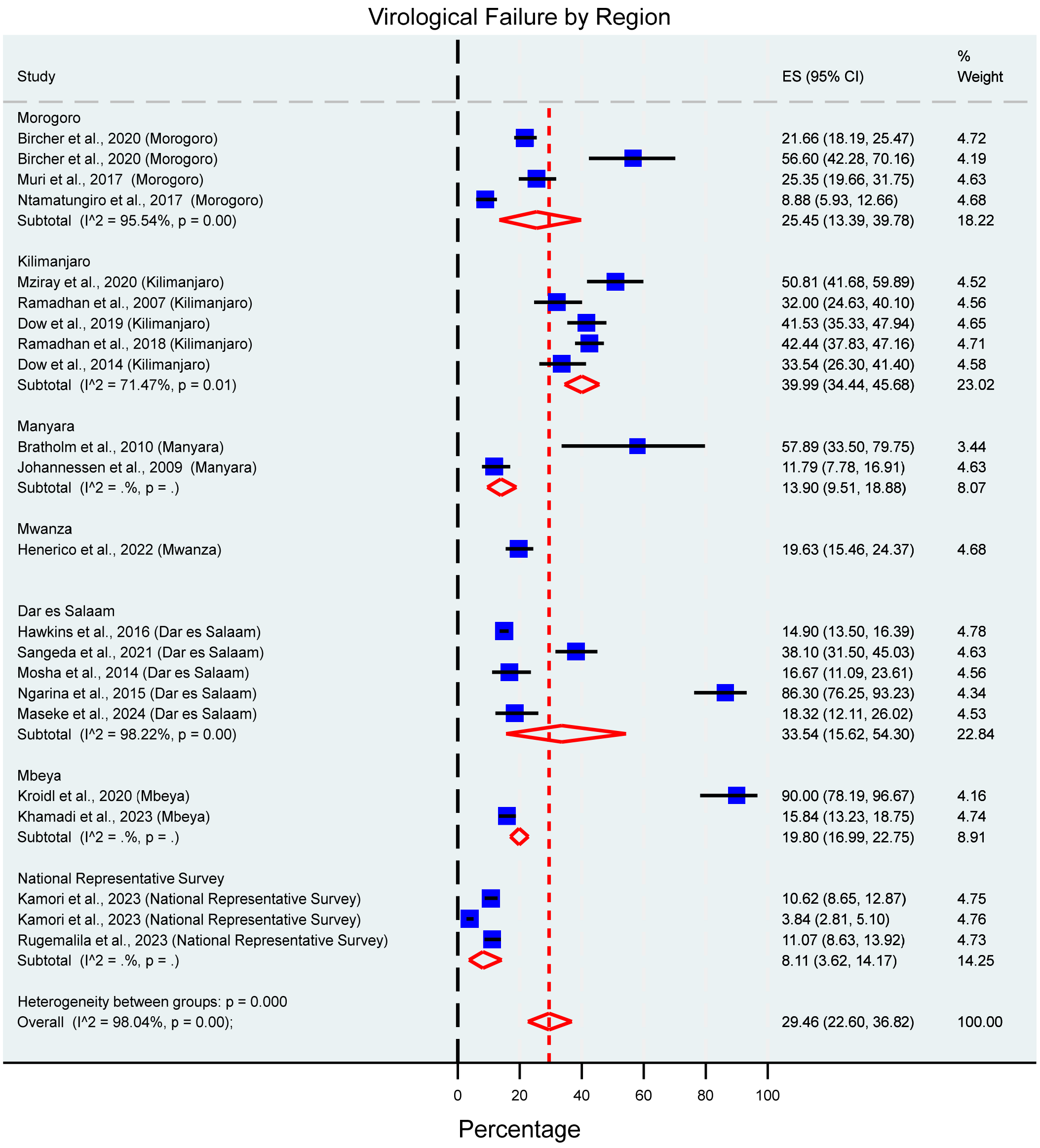

Figure S2. Prevalence of virological failure based on regions.

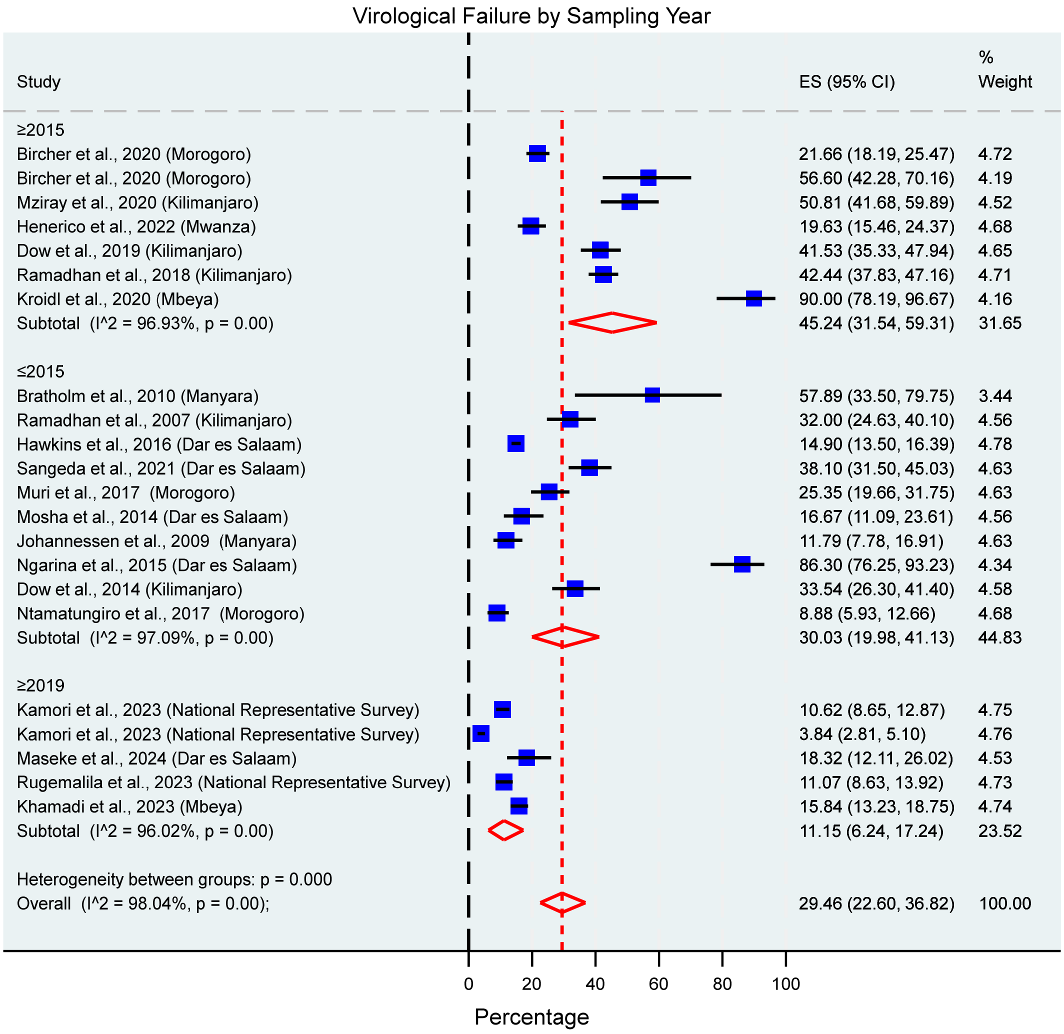

Figure S3. Prevalence of virological failure based on sampling years.

Subgroup analyses for Drug Resistance Mutations

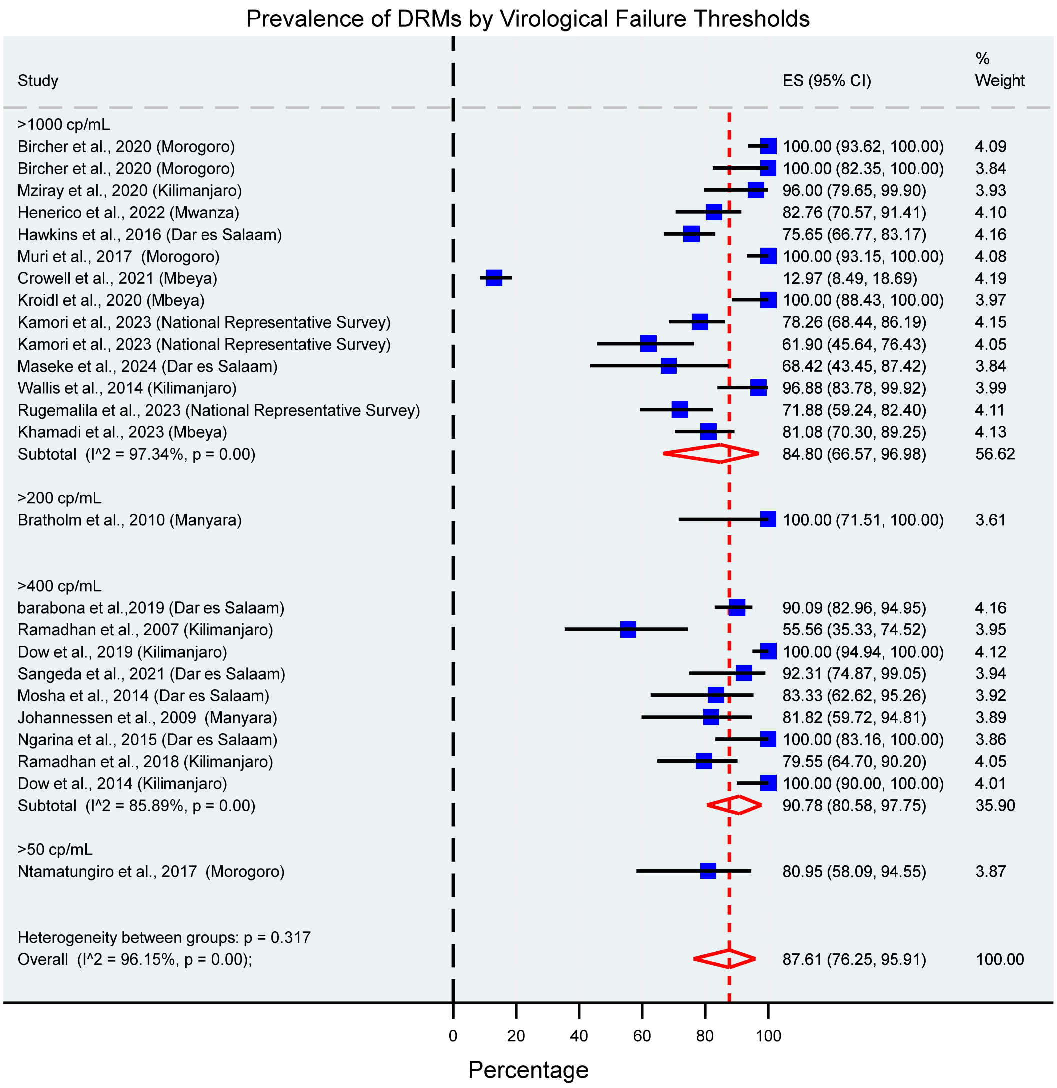

Figure S4. Prevalence of drug resistance mutations based on different virological failure thresholds as defined by the included studies.

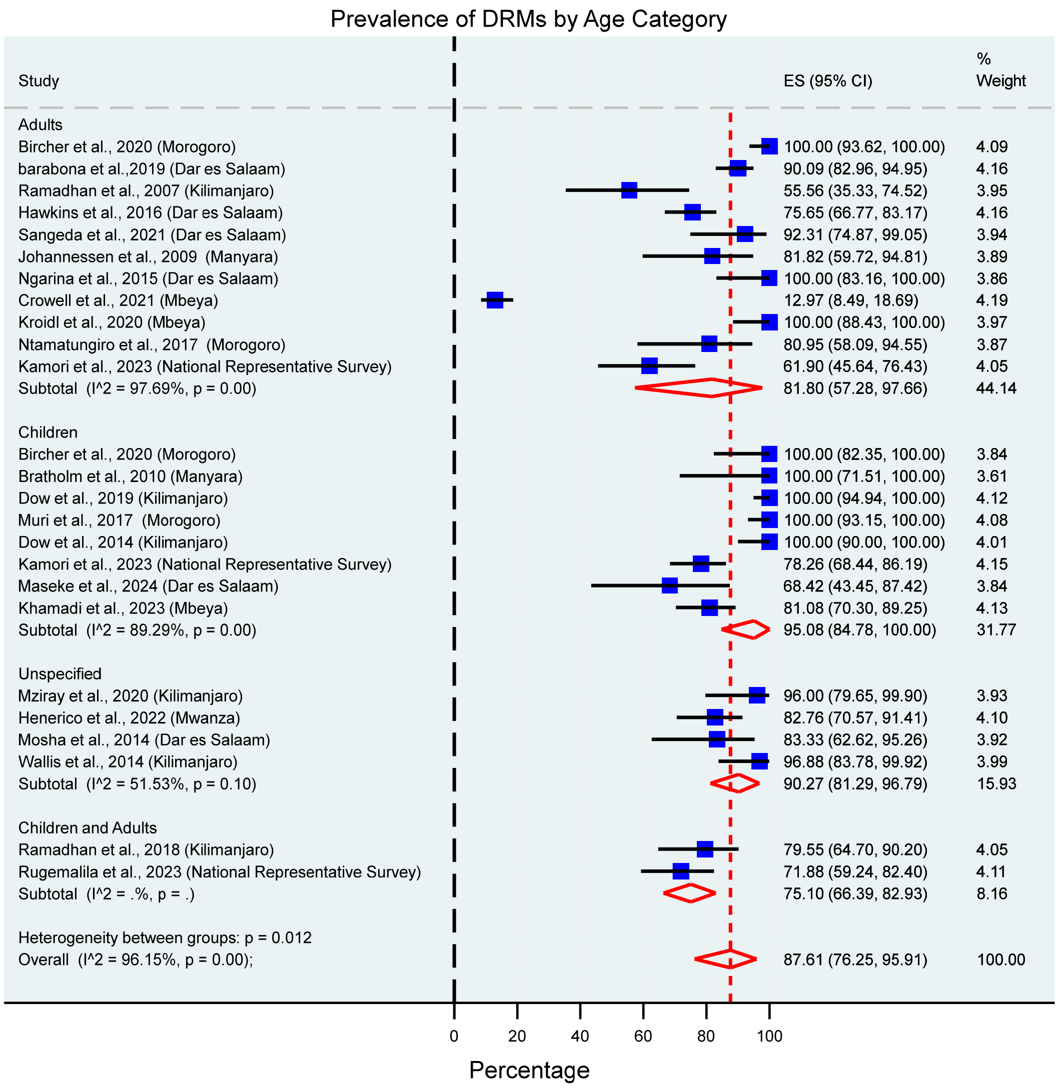

Figure S5. Prevalence of drug resistance mutations based on age categories

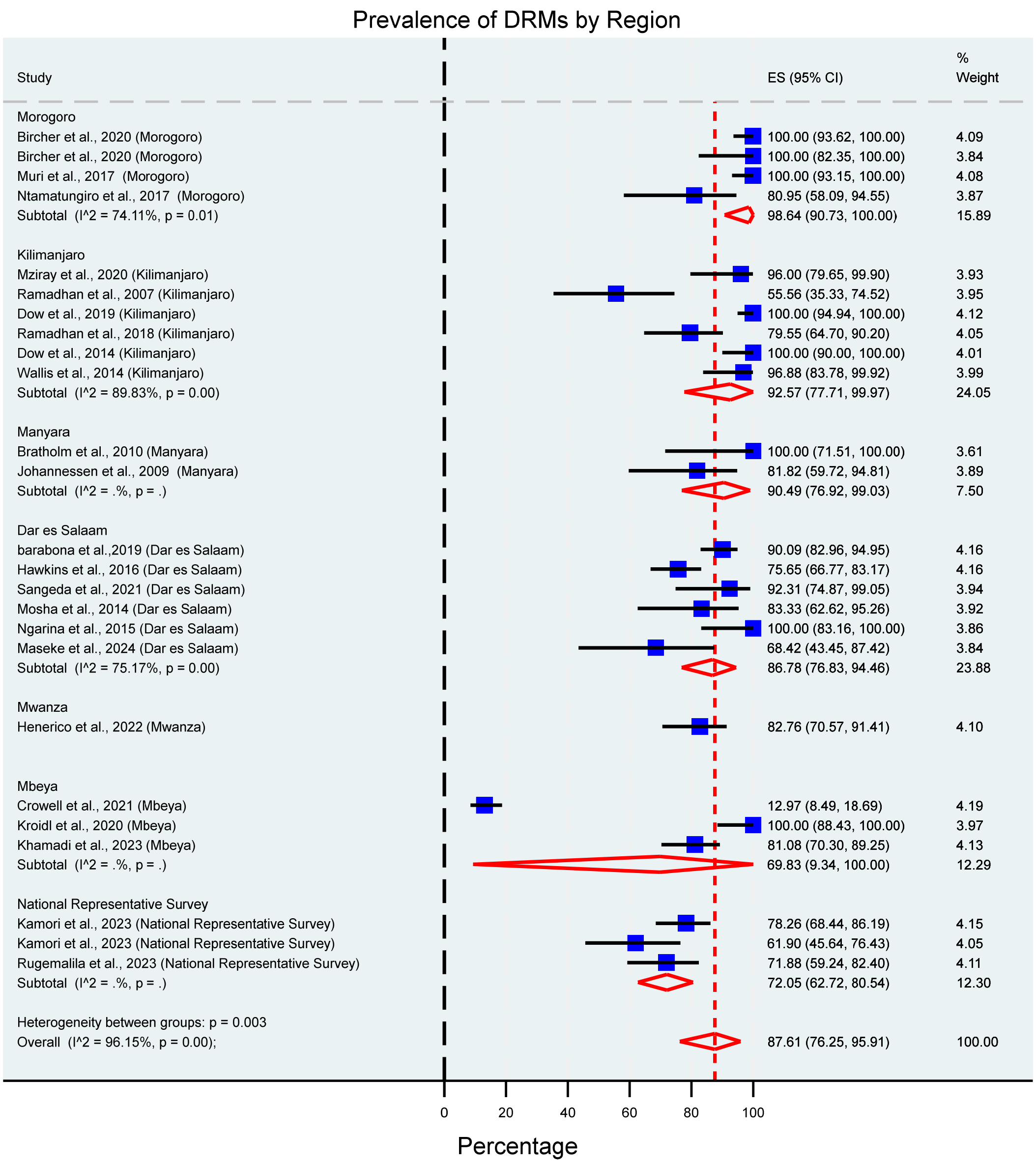

Figure S6. Prevalence of drug resistance mutations based on the region where the study was conducted.

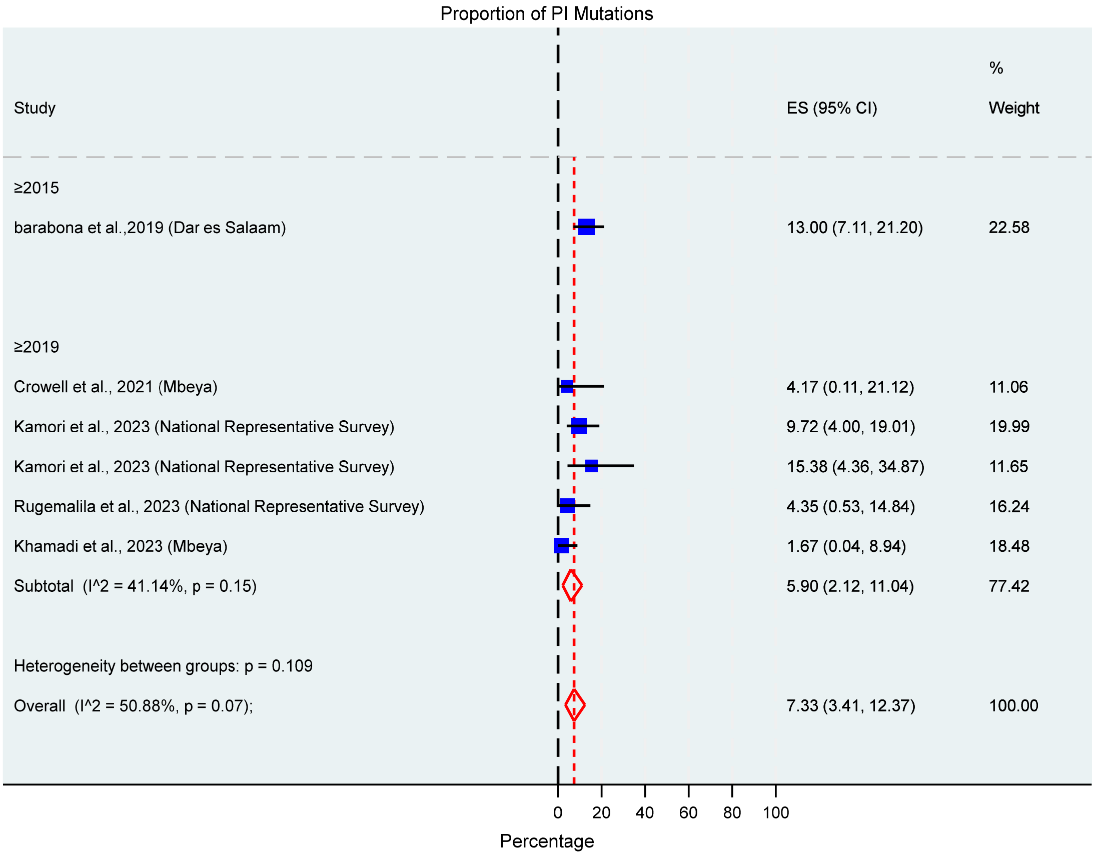

Figure S7. Proportion of PI mutations.

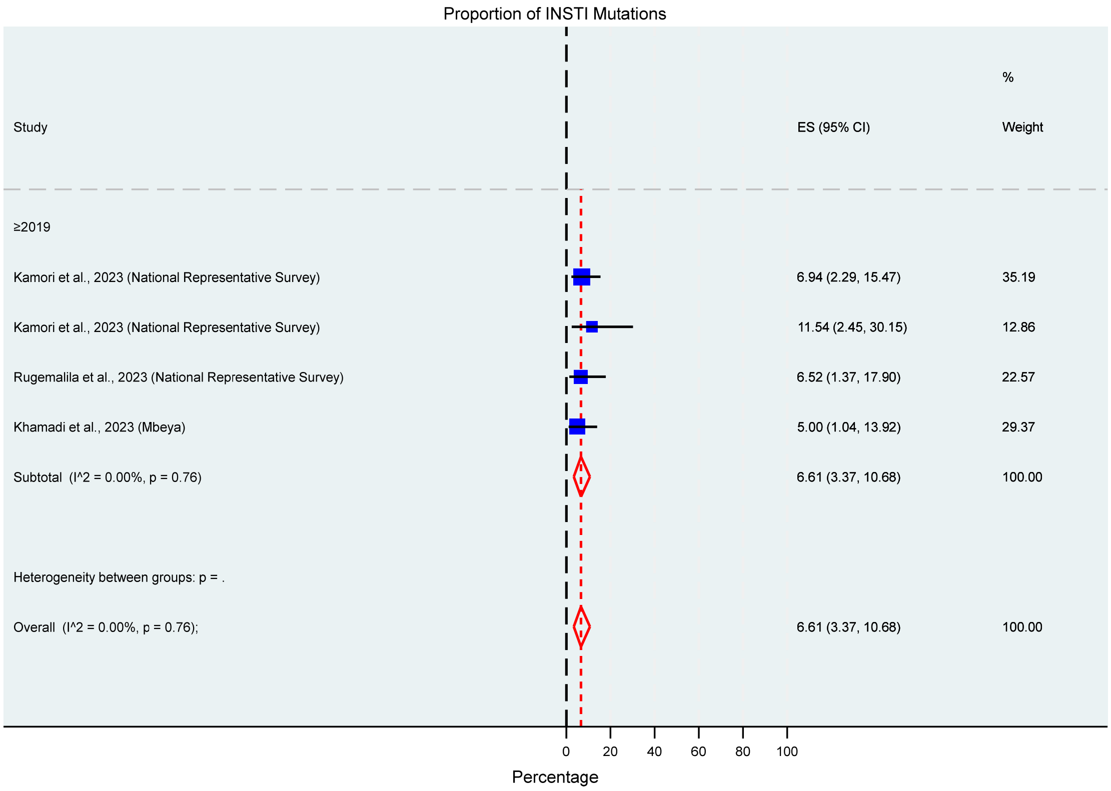

Figure S8. Prevalence of INSTI mutations.

Literature search

Table S1: Literature Search strategies for the different databases.

Database	Search Strategy
Medline through PubMed	(("drug resistance"[MeSH Terms] OR ("drug"[All Fields] AND "resistance"[All Fields]) OR "drug resistance"[All Fields]) AND ("mutate"[All Fields] OR "mutated"[All Fields] OR "mutates"[All Fields] OR "mutating"[All Fields] OR "mutation"[MeSH Terms] OR "mutation"[All Fields] OR "mutations"[All Fields] OR "mutation s"[All Fields] OR "mutational"[All Fields] OR "mutator"[All Fields] OR "mutators"[All Fields])) OR ("drug resistance"[MeSH Terms] OR ("drug"[All Fields] AND "resistance"[All Fields]) OR "drug resistance"[All Fields]) OR (("art"[MeSH Terms] OR "art"[All Fields]) AND ("resist"[All Fields] OR "resistance"[All Fields] OR "resistances"[All Fields] OR "resistant"[All Fields] OR "resistants"[All Fields] OR "resisted"[All Fields] OR "resistence"[All Fields] OR "resistences"[All Fields] OR "resistent"[All Fields] OR "resistibility"[All Fields] OR "resisting"[All Fields] OR "resistive"[All Fields] OR "resistively"[All Fields] OR "resistivities"[All Fields] OR "resistivity"[All Fields] OR "resists"[All Fields]))) AND (("hiv 1"[MeSH Terms] OR "hiv 1"[All Fields] OR "hiv 1"[All Fields]) AND ("subtype"[All Fields] OR "subtyped"[All Fields] OR "subtypes"[All Fields] OR "subtyping"[All Fields] OR "subtypings"[All Fields]))) OR (("hiv"[MeSH Terms] OR "hiv"[All Fields]) AND ("subtype"[All Fields] OR "subtyped"[All Fields] OR "subtypes"[All Fields] OR "subtyping"[All Fields] OR "subtypings"[All Fields])) OR ("hiv"[MeSH Terms] OR "hiv"[All Fields])) AND ("tanzania"[MeSH Terms] OR "tanzania"[All Fields] OR "tanzania s"[All Fields])
Embase	((((('drug resistance'/exp OR ('drug' AND 'resistance') OR 'drug resistance') AND ('mutate' OR 'mutated' OR 'mutates' OR 'mutating' OR 'mutation'/exp OR 'mutation' OR 'mutations' OR 'mutation s' OR 'mutational' OR 'mutator' OR 'mutators')) OR ('drug resistance'/exp OR ('drug' AND 'resistance') OR 'drug resistance') OR (('art'/exp OR 'art') AND ('resist' OR 'resistance' OR 'resistances' OR 'resistant' OR 'resistants' OR 'resisted' OR 'resistence' OR 'resistences' OR 'resistent' OR 'resistibility' OR 'resisting' OR 'resistive' OR 'resistively' OR 'resistivities' OR 'resistivity' OR 'resists'))) AND (('Human immunodeficiency virus 1'/exp OR 'hiv 1' OR 'hiv 1') AND ('subtype' OR 'subtyped' OR 'subtypes' OR 'subtyping' OR 'subtypings'))) OR (('Human immunodeficiency virus'/exp OR 'hiv') AND ('subtype' OR 'subtyped' OR 'subtypes' OR 'subtyping' OR 'subtypings')) OR ('Human immunodeficiency virus'/exp OR 'hiv')) AND ('Tanzania'/exp OR 'tanzania' OR 'tanzania s')
CINAHL	HIV Patient* OR "People living with HIV" OR "Persons living with HIV" OR "HIV-positive person*" OR "HIV-infected patient*" OR "Children AND HIV" OR "Adolescent* AND HIV" OR "HIV-infected women" OR "HIV-infected" OR "human immunodeficiency virus-infected children" OR "HIV-infected children" OR "Antiretroviral therap*" OR "Combined Antiretroviral Therap* " OR "Antiretroviral treatment*" OR "Combination Therap*" OR "Highly Active Antiretroviral Therap*" OR "protease inhibitor-based antiretroviral" OR "long-term antiretroviral treatment"<br><br>"drug* resistance-associated mutation" OR "acquired drug resistance-associated mutation" OR "clinical and virological response" OR "primary resistance" OR

	"Virological failure" OR "HIV-1 drug resistance mutation*" OR "acquired HIV-1 drug resistance mutation*" OR "HIV-1 acquired drug resistance mutation*" OR "HIV-1 drug resistance mutation*" OR "Antiretroviral drug resistance" OR "HIV-1 pol diversity" OR "HIV drug resistance" OR "HIV resistance" OR "Resistance associated mutation*" OR "Drug resistance mutation*" OR "Antiretroviral resistance" OR "HIV drug resistance mutation*" OR "Acquired Drug Resistance" OR clinical OR virological OR "Prevalence and Pattern*" OR Prevalence OR pattern*<br><br>Arusha OR Dar-es-Salaam OR Dodoma OR Geita OR Iringa OR Kagera OR Katavi OR Kigoma OR Kilimanjaro OR Lindi OR Manyara OR Mara OR Mbeya OR Morogoro OR Mtara OR Mwanza OR Njombe OR Pemba OR Pwani OR Rukwa OR Ruvuma OR Singida OR Shinyanga OR Simiyu OR Songwe OR Tabora OR Tanga OR Unguja OR "Urban Tanzania" OR "Rural Tanzania" OR Zanzibar OR Tanzania OR Tanzani OR "Southern Tanzania" OR "North-Western Tanzania"

Sensitivity Analysis Results

Table S2. Sensitivity analysis results for virological failure

**Omitted Study**	**Effect size**	**95% confidence interval**	**P-Value**
Bircher et al., 2020 (Morogoro)	0.302	0.207-0.397	0.000
Bircher et al., 2020 (Morogoro)	0.286	0.196-0.377	0.000
Mziray et al., 2020 (Kilimanjaro)	0.287	0.196-0.378	0.000
Bratholm et al., 2010 (Manyara)	0.290	0.199-0.381	0.000
Henerico et al., 2022 (Mwanza)	0.303	0.208-0.398	0.000
Ramadhan et al., 2007 (Kilimanjaro)	0.297	0.202-0.392	0.000
Dow et al., 2019 (Kilimanjaro)	0.292	0.198-0.385	0.000
Hawkins et al., 2016 (Dar es Salaam)	0.306	0.212-0.400	0.000
Sangeda et al., 2021 (Dar es Salaam)	0.294	0.199-0.388	0.000
Muri et al., 2017 (Morogoro)	0.300	0.205-0.395	0.000
Mosha et al., 2014 (Dar es Salaam)	0.304	0.210-0.398	0.000
Johannessen et al., 2009 (Manyara)	0.306	0.213-0.400	0.000
Ngarina et al., 2015 (Dar es Salaam)	0.267	0.191-0.344	0.000
Ramadhan et al., 2018 (Kilimanjaro)	0.291	0.197-0.385	0.000
Kroidl et al., 2020 (Mbeya)	0.270	0.191-0.349	0.000
Dow et al., 2014 (Kilimanjaro)	0.296	0.202-0.391	0.000
Ntamatungiro et al., 2017 (Morogoro)	0.308	0.215-0.400	0.000
Kamori et al., 2023 (National Representative Survey)	0.307	0.214-0.400	0.000
Kamori et al., 2023 (National Representative Survey)	0.310	0.220-0.401	0.000
Maseke et al., 2024 (Dar es Salaam)	0.303	0.209-0.397	0.000
Rugemalila et al., 2023 (National Representative Survey)	0.307	0.214-0.400	0.000
Khamadi et al., 2023 (Mbeya)	0.305	0.211-0.399	0.000
theta	0.297	0.207-0.387	0.000

Table S3. Sensitivity analysis results for drug resistance mutations

**Omitted Study**	**Effect size**	**95% confidence interval**	**P-Value**
Bircher et al., 2020 (Morogoro)	0.808	0.708-0.908	0.000
Bircher et al., 2020 (Morogoro)	0.812	0.713-0.910	0.000
Mziray et al., 2020 (Kilimanjaro)	0.812	0.713-0.912	0.000
Bratholm et al., 2010 (Manyara)	0.813	0.715-0.912	0.000
barabona et al.,2019 (Dar es Salaam)	0.812	0.711-0.914	0.000
Henerico et al., 2022 (Mwanza)	0.817	0.716-0.918	0.000
Ramadhan et al., 2007 (Kilimanjaro)	0.826	0.727-0.925	0.000
Dow et al., 2019 (Kilimanjaro)	0.807	0.707-0.907	0.000
Hawkins et al., 2016 (Dar es Salaam)	0.821	0.719-0.923	0.000
Sangeda et al., 2021 (Dar es Salaam)	0.813	0.714-0.913	0.000
Muri et al., 2017 (Morogoro)	0.808	0.709-0.908	0.000
Mosha et al., 2014 (Dar es Salaam)	0.817	0.717-0.916	0.000
Johannessen et al., 2009 (Manyara)	0.817	0.717-0.917	0.000
Ngarina et al., 2015 (Dar es Salaam)	0.811	0.712-0.910	0.000
Crowell et al., 2021 (Mbeya)	0.853	0.794-0.912	0.000
Ramadhan et al., 2018 (Kilimanjaro)	0.818	0.718-0.919	0.000

**Omitted Study**	**Effect size**	**95% confidence interval**	**P-Value**
Kroidl et al., 2020 (Mbeya)	0.810	0.711-0.909	0.000
Dow et al., 2014 (Kilimanjaro)	0.810	0.710-0.909	0.000
Ntamatungiro et al., 2017 (Morogoro)	0.817	0.718-0.917	0.000
Kamori et al., 2023 (National Representative Survey)	0.819	0.718-0.921	0.000
Kamori et al., 2023 (National Representative Survey)	0.826	0.726-0.925	0.000
Maseke et al., 2024 (Dar es Salaam)	0.821	0.722-0.920	0.000
Wallis et al., 2014 (Kilimanjaro)	0.811	0.712-0.911	0.000
Rugemalila et al., 2023 (National Representative Survey)	0.822	0.721-0.923	0.000
Khamadi et al., 2023 (Mbeya)	0.818	0.716-0.919	0.000
theta	0.817	0.720-0.914	0.000
